# A Tritordeum-Based Diet for Female Patients with Diarrhea-Predominant Irritable Bowel Syndrome: Effects on Abdominal Bloating and Psychological Symptoms

**DOI:** 10.3390/nu15061361

**Published:** 2023-03-10

**Authors:** Giuseppe Riezzo, Laura Prospero, Antonella Orlando, Michele Linsalata, Benedetta D’Attoma, Antonia Ignazzi, Gianluigi Giannelli, Francesco Russo

**Affiliations:** 1 Functional Gastrointestinal Disorders Research Group, National Institute of Gastroenterology IRCCS “Saverio de Bellis”, 70013 Castellana Grotte, Italy; 2 Scientific Direction, National Institute of Gastroenterology IRCCS “Saverio de Bellis”, 70013 Castellana Grotte, Italy

**Keywords:** abdominal bloating, diet, irritable bowel syndrome, nutritional profile, psychological profile, gastrointestinal symptom profile, tritordeum

## Abstract

Most female patients with irritable bowel syndrome (IBS) complain of abdominal bloating rather than abdominal pain and diarrhea. The higher incidence in women could be due to the so-called dysfunctional gas handling. Since diet seems the most effective and durable strategy for managing IBS symptoms, we aimed to evaluate the effects of a 12 week diet based on a relatively new cereal, Tritordeum (TBD), on gastrointestinal (GI) symptoms, anthropometric and bioelectrical impedance parameters, and psychological profiles in 18 diarrhea-predominant IBS (IBS-D) female patients with abdominal bloating as the dominant symptom. The IBS Severity Scoring System (IBS-SSS), the Symptom Checklist-90 Revised, the Italian version of the 36-Item Short-Form Health Survey, and the IBS-Quality of Life questionnaire were administered. The TBD reduces the IBS-SSS “Intensity of abdominal bloating” with a concomitant improvement in the anthropometric profile. No correlation was found between “Intensity of abdominal bloating” and “Abdominal circumference”. Anxiety, depression, somatization, interpersonal sensitivity, and phobic and avoidance manifestations were significantly reduced after TBD. Lastly, anxiety was correlated with “Intensity of abdominal bloating”. Overall, these results suggest the possibility of lowering abdominal bloating and improving the psychological profile of female IBS-D patients using a diet based on an alternative grain such as Tritordeum.

## 1. Introduction

Irritable bowel syndrome (IBS) is a functional gastrointestinal (GI) disorder (FGID) with a high prevalence. It affects 7–21% of the world population, with a female-to-male ratio of 2–2.5:1 in terms of those who seek medical care [1]. However, this female prevalence can differ depending on the clinical setting in relation to race, geography, community base, differences in access to health care, cultural factors, or help-seeking behavior [2]. It is a chronic condition with fluctuations over time, characterized by abdominal pain and altered bowel habits. Based on the prevalent stool characteristics, it can be categorized as IBS with predominant diarrhea (IBS-D), IBS with predominant constipation (IBS-C), and mixed IBS (IBS-M) [3].

This syndrome has a multifactorial genesis involving genetic, psychological, and environmental factors, inducing sensory, motor, immunological, and digestive alterations [4]. A close link between GI symptoms and the psychological profile is undeniable. In fact, despite the absence of a well-defined psychopathological diagnosis, IBS patients may suffer from anxiety and depression, along with higher levels of somatization compared to healthy subjects [4]. Such alterations can have clear repercussions on the quality of life (QoL) and work activities of affected individuals with high social burdens [5].

A high percentage of patients, ranging from 66% to 90%, mostly women, complain of abdominal bloating and distension rather than abdominal pain and diarrhea [6]. Abdominal bloating and distension may occur independently, but frequently coexist and even coincide with other FGIDs [7]. Abdominal bloating is the subjective sensation of trapped gas inside the bowel or the feeling of pressure without visible distension. In contrast, abdominal distension is the objective manifestation of increased abdominal circumference [7]. Female patients usually describe this phenomenon as “like a balloon” or “like I am pregnant” [8].

The causes of abdominal bloating and distension appear to be the concomitant presence of abnormal gas content in the bowel, alterations in intestinal microbiota composition, impairment of intestinal motility, and visceral sensitivity [9]. It is the relatively recent acquisition of so-called "minimal inflammation" in IBS patients that contributes, together with immune alterations and dysbiosis, to the onset of IBS symptomatology. The minimal inflammation can cause sensitization of enteric neurons, which can be longstanding after the inflammation resolves, as demonstrated in animal models mimicking FGID [10]. In addition, food intolerances, from single foods or categories (e.g., carbohydrates and cereals) to a more complex group of substances identified by the acronym FODMAPs (fermentable oligosaccharides, disaccharides, monosaccharides, and polyols), have to be considered as playing an active role [11].

The link between nutrition and GI symptoms in IBS patients is well established. The elimination diet seems to produce the most effective and durable effects for managing IBS symptoms. Beyond a pharmaceutical approach, great interest has been aroused by the possibility of managing this condition by adopting particular nutritional regimens [12]. Our group recently published a series of papers about the beneficial impact of drastically reducing FODMAPs from the diet of IBS-D patients in terms of ameliorated GI symptom profiles along with positive modifications of the intestinal permeability, mucosal integrity, immunological profile, and dysbiosis [13].

In this framework, much effort has also been devoted to evaluating the effects of foods based on a relatively new cereal called Tritordeum. This cereal derives from the hybridization of durum wheat and wild barley. Initially cultivated in Spain, it is now grown in Apulia, a region in southern Italy [14].

Interestingly, this cereal does not need much care and thrives even in adverse conditions. Tritordeum has lower levels of gliadin, carbohydrates, and fructans and higher fiber, protein, and antioxidant content than classical wheat. Although it is unsuitable for celiac patients, it may be helpful for subjects with non-celiac type wheat sensitivity (NCWS) or those IBS-D patients who benefit from removing wheat from the diet because of the onset of symptoms such as abdominal pain and bloating following its intake [15]. In addition, compared with the low-FODMAPs diet (LFD), a diet based on Tritordeum products (TBD) seems closer to Italians’ eating habits and more accessible to follow than other dietetic regimens [16]. Based on these premises, we aimed to investigate, in female IBS-D patients not responding to standard drug therapies and complaining of a symptom profile characterized by the presence of abdominal bloating as a dominant symptom, the effects of the TBD on symptom relief, together with possible modifications in the anthropometric, nutritional, and psychological profiles.

## 2. Materials and Methods

### 2.1. Subject Recruitment

Patients suffering from IBS-D in accordance with Rome III–IV criteria [17] were recruited from March 2022 to November 2022 from among the outpatients of the FGID Research Unit-National Institute of Gastroenterology, IRCCS “Saverio de Bellis”.

Patients (18–65 years) underwent GI visits and physical examinations. They had to complain of symptoms resembling IBS-D (present for at least two weeks). The Gastrointestinal Symptom Rating Scale (GSRS) questionnaire [18] was administered during the visit as an initial screening.

Blood tests for liver and thyroid function, reactive protein C, FOBT on three determinations, stool culture, and a stool test for parasites, performed within the previous three months, were required for all patients. Recent gastroscopy and colonoscopy with biopsy samples to exclude microscopic colitis were also needed. 

Criteria of exclusion were: pregnancy, a diagnosis of metabolic or endocrine disorders, hepatic, renal, or cardiovascular disease, constipation, post-infectious IBS, giardiasis, previous abdominal surgery, fever, intense physical activity, secondary causes of intestinal atrophy, a history of malignancy; antibiotic therapy or probiotic agents and other medications known to cause abdominal pain, assumption of selective serotonin reuptake inhibitors (SSRIs) and other antidepressant drugs, and no therapies for IBS in the last two weeks before evaluation. Celiac markers (tissue transglutaminase and anti-endomysium antibodies) were evaluated. Before entering the study, patients should have refrained from diets that excessively restrict certain nutrients (e.g., LFD, gluten-free, and vegan diets).

Reasons for withdrawal from the study were recorded in the case report form. They could include: adverse events (specified), ineligibility to continue the study, removal of consent, lost to follow-up, and other causes.

All subjects were compliant and willing to participate in the study. They gave their informed consent for blood tests and clinical data collection. This study was part of a research project approved by the local scientific committee and the institutional ethics committee of the IRCCS Oncological Hospital of Bari “Giovanni Paolo II Cancer Institute”, Bari, Italy (Prot. No. 143/EC of 23 March 2022, registered at http://www.clinicaltrials.gov; NCT05307185, last access date 11 April 2022). 

### 2.2. Study Design

**Figure 1:**
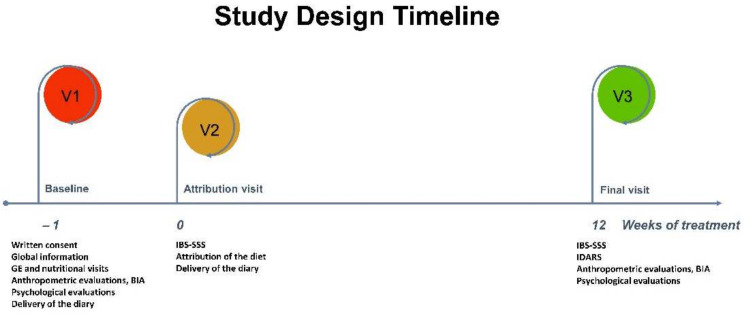
The study design timeline. GE: Gastrointestinal examination; BIA: Bioelectrical impedance analysis; IBS-SSS: IBS Symptom Severity Scale; and IDARS: IBS diet-adherence report scale.

The study consisted of three visits (Figure 1):

V1: At baseline, patients underwent a gastroenterological visit, received verbal and written information on the study, and provided informed consent. They were advised that the study was intended to evaluate the ability of TBD to reduce IBS symptoms and that it was to be followed for 12 weeks. Qualified nutritionists interviewed the recruited subjects to evaluate their lifestyle, eating habits, physical activity, physiological conditions, and concomitant pathologies. In addition, a bioelectrical impedance analysis (BIA) was performed, and the anthropometric parameters were evaluated. The patients eligible for the study were invited to consume their usual diet and fill out a daily diary of their food habits, also recording their stool characteristics (according to the Bristol stool form chart) [19], intestinal habits, medications, and physical activity to estimate their daily energy intake and consumption until the next visit (V2);

V2: One week after the first visit, patients returned to the clinic to complete the IBS Symptom Severity Scale (IBS-SSS) [20]. An IBS-SSS symptom total score of at least 75 was required to be included in the study. In addition, inclusion and exclusion criteria, and dietary habits, were revised by examining the daily dietary diaries recorded during the seven days before V2. Patients participating in the study were then asked to follow their personalized TBD and keep a daily diary until the end of the dietary intervention, as they did for V1. Patients were invited to abstain from alcohol or engage in vigorous physical activity. Patients kept food diaries to assess their energy intake and expenditure during the study period. Energy intake refers to the calorie intake of food and drinks per unit of time (24 h). Energy expenditure is the total energy expended by the organism in a unit of time (24 h) to maintain its structural and functional properties and perform physical activities. Along with the description of physical activity and its duration, the diary contained the quantities (in grams) and descriptions of foods eaten each day for breakfast, lunch, dinner, and snacks. Psychological characteristics and stress levels were assessed by administering psychometric instruments described in detail below;

V3: Twelve weeks after starting the diet, symptom and diet questionnaires completed in the previous days were collected. Patients received the IBS-SSS questionnaire and the IBS diet-adherence report scale (IDARS). Lastly, the BIA, anthropometric measurements, and psychological questionnaires were repeated.

### 2.3. Assessment of Anthropometric and BIA Parameters

The following anthropometric parameters were evaluated: height, weight, body mass index (BMI), mid-upper arm, shoulder, abdominal, waist, and hip circumferences. The weight and height for calculating the BMI (kg/m^2^) were measured by a SECA mod. 700 mechanical column scale and a mod. 220 SECA altimeter (INTERMED S.R.L., Milan, Italy). The abdominal and waist circumferences were calculated using a mod. 201 SECA tape measure. 

All patients undergoing BIA had fasted for at least 4 h and had not ingested alcohol or experienced intense physical activity in the previous 12 h.

The BIA assesses the resistance (Rz) and reactance (Xc) of human tissue by injecting a constant (800 µA) sinusoidal current at 50 kHz. Measurements were performed using the same equipment (BIA 101 BIVA PRO, Akern SRL, Pontassieve, Italy) under strictly standardized conditions according to the guidelines of the European Society for Parenteral and Enteral Nutrition (ESPEN) [21]. Phase angle (PhA, calculated as the arctangent of Xc/Rz ratio), body cell mass (BCM) and body compartments, i.e., fat-free mass (FFM), fat mass (FM), total body water (TBW), and extracellular water (ECW) were calculated directly from Rz and Xc by medically validated algorithms using dedicated software (Bodygram PLUS Software v. 1.0, Akern SRL, Pontassieve, Italy).

### 2.4. Psychological Questionnaires

#### 2.4.1. IBS Quality of Life Questionnaire (IBS-QoL)

The IBS-QoL only assesses the IBS patients’ QoL. The questionnaire has 30 items divided into nine subscales: total score, dysphoria, interference with activity, body image, health worry, food avoidance, social reaction, sexual concerns, and relationship. Raw scores are converted to scale scores from 0 to 100, with higher scores indicating better quality of life [22].

#### 2.4.2. Thirty-Six Item Short-Form Health Survey (SF-36)

The SF-36 is a short questionnaire aimed at evaluating the health condition and investigating the QoL of patients, independently from the complained disease. The subscales and the global indexes are organized so that the higher the score, the better the health status. The first three sub-scales describe physical health (physical activity and limitations of role-specific activities due to physical problems and physical pain). The intermediate two subscales describe global health in general (general health and vitality). The last three subscales describe those aspects related to psychological and emotional health (limitations in social activity and restrictions of role-specific activities caused by mental health or emotional problems). An additional unscaled single item reports changes in the respondent’s health over the past year. For each variable, scores were coded, summed, and transformed on a scale from 0 (worst possible health state) to 100 (best imaginable health state) [23].

#### 2.4.3. Symptom Checklist-90-Revised (SCL-90-R) 

The SCL-90-R is a commonly applied tool for self-reporting symptoms [24]. The SCL-90-R evaluates a wide range of psychopathological symptoms, with nine main symptom dimensions (somatization, obsessive-compulsive, interpersonal sensitivity, depression, anxiety, hostility, phobic anxiety, paranoid ideation, and psychoticism) and three global indexes. The Global Severity Index (GSI) was the only one used in the study since it best represents the intensity of psychological distress that subjects perceive. The raw score is converted to a T score; a score equal to or higher than 63 is considered a clinically significant symptom. 

#### 2.4.4. Zung’s Self-Rating Anxiety Scale (SAS)

The Zung SAS is a self-report scale comprising 20 items that investigate different types of anxiety symptoms, both psychological and somatic. Responses are presented on a 4-point scale. Raw scores range from 20 to 80 [25]. The higher the standard score, the more serious the symptom.

#### 2.4.5. Zung’s Self-Rating Depression Scale (SDS)

The SDS consists of 20 items and is based on the diagnostic criteria for depression. Subjects consider each item concerning how they have felt in the past few days through a 4-point Likert scale. The sum of the raw scores ranges from 20 to 80 [26]. The higher the standard score, the more serious the symptom.

#### 2.4.6. Psychophysiological Questionnaire (QPF/R) 

The QPF/R is part of the Cognitive Behavioral Assessment 2.0 (CBA 2.0), a test battery that provides a general overview of psychological problems in the individual and social domains. It consists of 10 schedules, of which the QPF is contained in the sixth one. The QPF/R evaluates stress and psychophysiological disorders [27].

### 2.5. IBS Severity Scoring System (IBS-SSS)

The IBS-SSS was administered to evaluate the GI symptom profile. This validated questionnaire consists of five items: “Intensity of abdominal pain”, “Frequency of abdominal pain”, “Severity of abdominal bloating”, “Dissatisfaction with bowel habit”, and “Interference with life in general”, scoring from 0 to 500. As specified in the appendix of the paper from Francis et al. about the validation of the IBS-SSS questionnaire, “abdominal distension” refers to “bloating, swollen or tight tummy”, so abdominal bloating was used to avoid misunderstanding [20]. The widely used cut-off of IBS-SSS scores was applied to evaluate the severity of IBS: >75–175 for mild IBS, 175–300 for moderate IBS, and >300 for severe IBS.

### 2.6. Characteristics of a Tritordeum-Based Diet (TBD)

The nutritional treatment was characterized by a balance of the energy ratio (a daily intake of 50% carbohydrates, 30% lipids, and 20% proteins) calculated using dedicated software (Nutrigeo Software 8.6.0.0, Progeo Medical, Centobuchi di Monteprandone, Italy). The daily energy intake and the intake (in kcal) of carbohydrates, lipids, and protein (in percent and weight), alcohol consumption (in percent), and dietary fiber (in grams) were also calculated. 

Overall, patients in the study received detailed instructions for their daily intake, which included three meals (breakfast, lunch, and dinner) and two snacks (morning and afternoon). 

All the food items (bread, pasta, “taralli” local salty biscuits, and breakfast biscuits) were prepared using Tritordeum flour by Intini Food (Putignano, Italy). The energy content, the chemical composition of Tritordeum flour and pasta, and the ingredients and preparation of Tritordeum-based foods used in the study have previously been described [15].

### 2.7. IBS Diet-Adherence Report Scale (IDARS)

IDARS consists of five questions on adherence to dietary treatment, with a score for each item ranging from one to five. A total score equal to or higher than 20 represents good diet adherence [28].

### 2.8. Statistical Analysis

The primary endpoint was the clinical response after 12 weeks of dietary intervention, as defined by a ≥50-point reduction in the IBS-SSS symptom severity total score. The estimated minimum sample size was 18 subjects. Based on a 45% dropout probability, at least 40 patients had to be recruited. Unless otherwise specified, all results were expressed as means ± SEM. The Wilcoxon matched-pairs signed rank test, a non-parametric test, was performed to compare pre- and post-treatment data because of the small number of patients studied and to avoid the assumption of normal distribution. A Spearman correlation analysis was performed to determine the putative correlations between symptoms, anthropometric measurements, and psychological scales. The statistical packages were Sigma Stat 11.0 (Systat Software, Inc., San Jose, CA, USA) and GraphPad Prism 5 (GraphPad Software, Inc., La Jolla, CA, USA). A *p*-value less than 0.05 was considered statistically significant.

## 3. Results

### 3.1. Patients’ Number and Response to Diet

Of the 40 female patients enrolled, 18 (44.72 ± 2.43 years) completed the study. The patients showed excellent adherence to TBD, as demonstrated by the total IDARS mean score, which was 22.39 ± 0.36, higher than the desired value of 20. The proportion of patients defined as responders (IBS-SSS reduction ≥ 50 score points) was 16/18 (88.88%).

### 3.2. Gastrointestinal (GI) Symptoms

Based on the cut-off previously reported, the patients presented an average total score of IBS-SSS classified as “moderate” at baseline, and after TBD, it dropped to a “mild” level (Table 1). The percentage reduction in the total score was −50.50%. Analyzing the individual items, the dominant symptom recorded at baseline was “Severity of abdominal bloating” and “Dissatisfaction with bowel habit”. At baseline, the patients presented only a slight alteration in stool consistency, as demonstrated by the Bristol stool form, despite an evident improvement in bowel habit satisfaction. It should be noted that the Bristol score passed from 5 to 4, which is considered normal, even if there was no significant difference from a statistical point of view. All the other single items showed a slight reduction of about 50% after TBD. “Abdominal pain frequency” reduced by −57.78%, the most dramatic decrease after TBD. “Interference on life in general” also decreased and will be described in detail with the psychological and QoL questionnaires. Lastly, there was found to be a positive correlation between “Intensity of abdominal pain” and “Intensity of abdominal bloating” (r = 0.73, *p* < 0.0001).

### 3.3. Anthropometric and BIA Parameters

Table 2 describes anthropometric and BIA data before and after TBD. Several anthropometric items significantly decreased (e.g., weight, BMI, anthropometric circumferences), along with some BIA parameters such as FM, FFM, TBW, and ECW. In detail, “Abdominal circumference” reduced from 95.65 ± 2.39 cm to 88.39 ± 2.30 cm (*p* = 0.0143). The “Abdominal circumference”, as well as the BMI, did not correlate to the IBS-SSS items “Severity of abdominal pain” and “Severity of abdominal bloating”. 

### 3.4. Psychological and QoL Profiles

**Figure 2:**
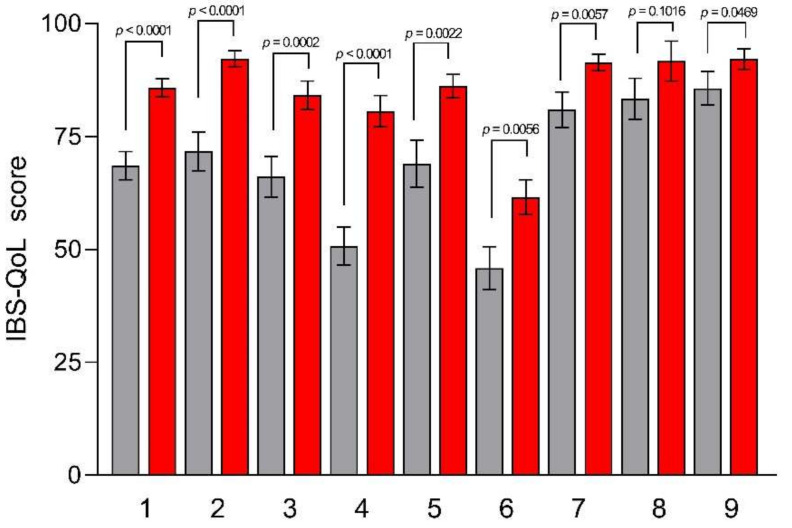
IBS-QoL subscale scores before and after the Tritordeum-based diet. Data are expressed as means ± SEM and analyzed by the Wilcoxon matched-pairs signed rank test. All differences were considered significant at *p* < 0.05. Subscales: (**1**) total score; (**2**) dysphoria; (**3**) interference with activity; (**4**) body image; (**5**) health worry; (**6**) food avoidance; (**7**) social reaction; (**8**) sexual concerns; and (**9**) relationship. Grey columns (Pre-diet), red columns (Post-diet).

Figure 2 provides an overview of the mean IBS-QoL total score and subscales at baseline and after TBD. The IBS-QoL mean total score at baseline was 68.54 ± 3.12 and increased significantly after TBD (mean total score 85.77 ± 1.97, *p* < 0.0001). Regarding IBS-QoL subscales, there was a statistically significant increase after TBD for all subscales, except for the “Sexual concerns” one.

**Figure 3:**
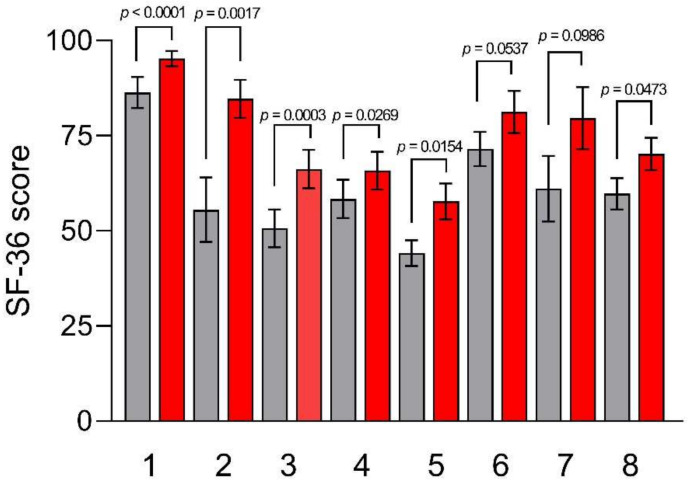
SF-36 subscale scores before and after the Tritordeum-based diet. Data are expressed as means ± SEM and analyzed by the Wilcoxon matched-pairs signed rank test. All differences were considered significant at *p* < 0.05. Subscales: (**1**) physical function; (**2**) role physical; (**3**) body pain; (**4**) general health; (**5**) vitality; (**6**) social functioning; (**7**) role emotional; and (**8**) mental health. Grey columns (Pre-diet), red columns (Post-diet).

Figure 3 describes the mean scores of the SF-36 subscales. At baseline, the lowest SF-36 score was for the role limitations due to physical problems subscale (55.56 ± 8.47), “Body pain” (50.67 ± 4.95), and “Vitality” (44.17 ± 3.34), while the highest scores were for “Physical and Social functioning” subscales (86.39 ± 4.08 and 71.53 ± 4.50, respectively). After TBD, all subscales had a statistically significant improvement, except for “Social functioning” and role limitations due to emotional problems.

**Figure 4:**
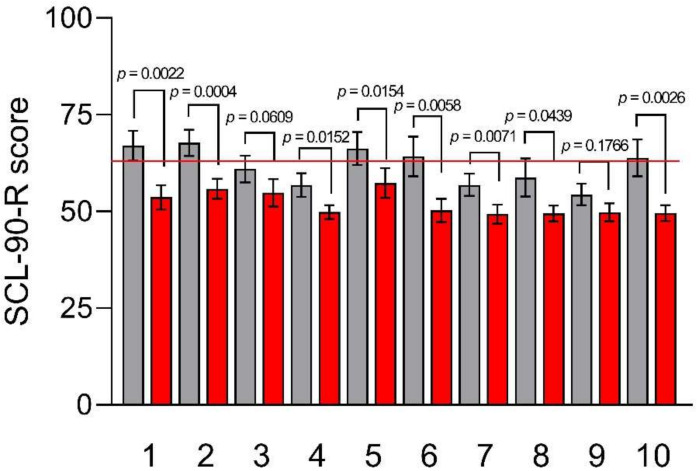
SCL-90-R subscale scores before and after the Tritordeum-based diet. Data are expressed as means ± SEM and analyzed by the Wilcoxon matched-pairs signed rank test. All differences were considered significant at *p* < 0.05. Subscales: (**1**) GSI; (**2**) somatization; (**3**) obsessive-compulsive; (**4**) interpersonal sensitivity; (**5**) depression; (**6**) anxiety; (**7**) hostility; (**8**) phobic anxiety; (**9**) paranoid ideation; and (**10**) psychoticism. The red line indicates the cut-off value (63). Grey columns (Pre-diet), red columns (Post-diet).

As regards the SCL-90-R, the results of which are illustrated in Figure 4, at baseline, the mean index GSI (67.06 ± 3.83), as well as the “Somatization” (67.78 ± 3.39), “Depression” (66.28 ± 4.23), “Anxiety” (64.28 ± 5.14), and “Psychoticism” (63.89 ± 4.76) subscales, were higher than the cut-off value. After TBD, all SCL-90-R subscale scores fall below the cut-off value, significantly reducing the “Somatization”, “Anxiety,” and “Psychoticism” subscale scores (55.89 ± 5.60 *p* = 0.0004, 50.28 ± 2.98 *p* = 0.0058, and 49.61 ± 2.05 *p* = 0.0026). 

Finally, the levels of anxiety, depression, and stress experienced by the patients were also assessed (SAS, SDS, and QPF/R). At baseline, the anxiety level was straddling low to moderate, while at the end of TBD it had significantly dropped 5 points (38.39 ± 0.89 vs. 33.39 ± 0.75, *p* < 0.0001). Regarding depression, at the start of the study, the mean score was placed in the low severity range. At the end of the treatment, it dropped further, with a statistically significant difference (38.83 ± 1.48 vs. 35.67 ± 1.03, *p* = 0.0137). Finally, the psychophysiological activation at baseline was particularly high (61.50 ± 1.65; cut-off 50). After TBD, there was a statistically significant decrease (*p* < 0.0001) in the score despite persisting above the cut-off levels (54.06 ± 1.87). A significant correlation was found between the level of anxiety, measured by SAS, and “abdominal bloating” (r = 0.47, *p* = 0.0038).

## 4. Discussion

Management of IBS patients should ensure lasting improvement in symptoms since these patients suffer from a pronounced QoL impairment, which causes a high economic burden and decreases productivity in the workplace. Our present results confirm that bloating may be the most bothersome symptom of female patients with IBS, even if the Rome criteria do not consider it part of the IBS definition. In addition, bloating is associated with other symptoms, such as abdominal pain and an altered psychological profile. Lastly, 12 weeks of TBD leads to a clear reduction of bloating with a concomitant improvement in the nutritional and psychological profiles and QoL. 

Beyond symptoms, TBD has been demonstrated to ameliorate a series of pathophysiological parameters in IBS-D patients, such as altered intestinal permeability, a pro-inflammatory immunological profile, and dysbiosis [15]. As reported elsewhere [15,16,29], the reduced contents of gliadin, fructans, and carbohydrates in this cereal seem responsible for the improved symptom profile. In the present study, our attention was focused on abdominal bloating. This symptom is present in about 30% of the general population, and in most cases, it is so severe as to compromise QoL (54%), or it requires medication (43%) [30].

Although the concepts of abdominal bloating and distension are often used interchangeably, it has recently been suggested to use abdominal bloating to describe the subjective sensation of intra-abdominal gas. In contrast, abdominal distension refers to the actual increase in abdominal circumference. Both abdominal bloating and distension are present in almost all IBS patients. The presence of abdominal bloating alone is common in IBS-D patients, while abdominal bloating associated with distension is usually present in IBS-C [31]. They can be part of the symptom profile of various FGIDs, from dyspepsia to IBS, even identifying a distinct group of patients grouped with the term “functional bloating” [32]. Some authors have also suggested considering a specific subgroup of IBS patients whose main characteristic is excessive gas production, abdominal bloating, and visible abdominal distension [33]. In contrast, others have proposed categorizing IBS subgroups according to the presence or absence of pain and abdominal bloating [34].

The causes of these symptoms are different, involving food intolerance, minimal inflammation, alterations of visceral sensitivity, and intestinal dysbiosis. These alterations increase the production of luminal gas, its altered passage and clearance, and cause excessive content of luminal liquids, which can be summarized in the so-called dysfunctional gas handling [35]. Excessive gas and liquid lead to distension of the colon wall and the consequent bloating. Causes could also include the influence of a psychological profile, although it is currently unclear whether this is the result or cause of more severe bloating symptoms in IBS patients [36]. 

This condition is typically gender-related and mainly present in women, probably due to hormonal factors, different pain thresholds, and colonic microbiota composition [37].

As regards hormonal profile, bloating is the most frequently reported symptom among patients with a common endocrine disorder, polycystic ovary syndrome (PCOS), and appears to be its primary predictor [38]. Bloating can be experienced perimenstrually by healthy women [39], although the mechanisms underlying the symptom might be different from those in other pathological situations. An association between IBS and sex hormonal status (menstrual cycle phase, pregnancy, menopause, and hormonal replacement therapy) has been recognized [40,41]. These sex hormones influence peripheral and central regulatory mechanisms involved in the pathophysiology of IBS, causing alterations in the stress response, visceral sensitivity and motility, intestinal barrier function, and immune activation of the intestinal mucosa. They also directly affect the gut microbiota and enteric nervous system [2]. Low-grade inflammation and related impaired intestinal permeability, specific conditions linked to IBS pathophysiology, at least in a subgroup of patients, can contribute to heightened visceral sensation and its onset along with pain [42]. Lastly, changes in gut microbiota could contribute to inducing such symptoms [43], even if attempts to modify intestinal bacterial populations by administering probiotics or antibiotics have produced conflicting or no results [44,45].

Unfortunately, pharmacological management is limited and ineffective; therefore, the most logical alternative is the dietary approach, with which psychological interventions could be associated. At baseline, our female IBS patients presented an IBS-SSS mean total score categorized as “moderate”, with abdominal bloating rather than abdominal pain as the dominant symptom. After 12 weeks of treatment, a significant and parallel reduction in abdominal bloating and pain occurred. Interestingly, the decline was precocious, already evident after the first month of diet. However, to uniformly compare the clinical and biochemical data, all the variables were evaluated at the start and end of treatment. The reduction after TBD was similar for both items. Particularly evident were the reduction in abdominal pain frequency and the dissatisfaction with bowel habits.

It is noteworthy to highlight the absence of significant changes in the Bristol stool score after TBD, even with the reduction in “bowel dissatisfaction”. This evidence indicates that the improvement in bowel habits is not a “simple” change in stool consistency involving other factors (e.g., the number and frequency of bowel movements, the effort to defecate, and unsuccessful attempts). 

Abdominal bloating was associated with significantly greater IBS symptoms, specifically pain severity. In fact, there was a significant correlation between the “Intensity of abdominal pain” and the “Intensity of abdominal bloating”. These data were in accordance with previous studies investigating the association of abdominal bloating with other symptoms in IBS patients [36,46]. At the same time, no correlation was found between the “Intensity of abdominal bloating/pain” and the anthropometric data on “Abdominal circumference”. These results demonstrate that the relationship between the two symptoms (bloating and distension) may depend on the severity of the abdominal bloating. Some visceromotor reflexes that modulate abdominal muscle tone are activated only at a certain threshold, creating a protrusion of normal abdominal content [47].

One could argue that a placebo effect may affect the symptom score. To refute this claim, we evaluated a series of anthropometric and nutritional indices that are certainly independent of this effect. 

The balanced and tailored TBD ensured an adequate distribution of calories and fiber over five meals, excluding foods the patient could be intolerant of (e.g., FODMAPs). At the end of treatment, the anthropometric indices were significantly reduced, particularly the abdominal circumference, even if it remained above the optimal value. Overall, these results confirmed the existence of a non-specific effect due to a personalized diet, as highlighted by the impact on both BMI and the BIA parameters.

The other aim of the present paper was to investigate the relationship between bloating and the psychological profile and QoL of IBS-D patients. Past studies about the putative relationship between bloating and psychological factors have produced inconsistent data. More recently, however, an association has been identified between abdominal bloating and significant psychological distress, particularly somatization and depression, which has been confirmed by several studies [9,48]. Anxiety, depression, and somatization scores are associated with higher pre- and post-prandial GI symptoms. More specifically, anxiety was associated with fullness and bloating, while depression was associated with abdominal pain. However, the most robust finding was for somatization, which was related to higher levels of all GI symptoms [49]. The results from that study are in line with ours, in which anxiety levels were found to be closely and significantly correlated with bloating.

Therefore, different psychological questionnaires (such as the IBS-QoL, SF-36, SCL-90-R, SAS, SDS, and QPF/R) were used. The obtained data clearly showed, at baseline, an altered psychological profile of our patients and the reduction of numerous subscales, particularly anxiety and depression, at the end of treatment. These results suggest the intriguing possibility of modulating the psychological profile of IBS patients using a nutritional approach. When GI symptoms attenuated and no longer constituted an obstacle in daily activities, a direct positive effect on QoL could be observed, as evidenced by the analysis of the total score and in the IBS-QoL subscales. The subject did not worry about the GI symptoms anymore, and her psychological profile improved. Dysphoria, along with the unpleasant feelings that characterize it (e.g., concerns for one’s health, avoidance behaviors for foods), is dramatically reduced. As a result, the reduction of avoidance and isolation behaviors, enacted due to the shame and embarrassment that IBS patients feel about their condition, induces an improvement in professional, relational, and personal life. 

The SF-36 also showed the same trend. The physical and emotional interference on daily functioning due to GI symptoms was reduced after the diet, resulting in better mental health conditions. More specifically, after TBD, the psychopathological risk improved, as evidenced by the results of the SCL-90-R. Thus, as the GI symptoms decreased, hostility, anxiety, depression, interpersonal sensitivity, phobias, and avoidance manifestations were significantly reduced. Somatization, whose scores improved significantly after TBD, also benefited from this treatment. It should be emphasized that somatization in IBS patients increases access to resources and health service assistance and decreases treatment response and compliance [50]. Overall, these results demonstrate the strong impact a specific diet can have on GI symptoms, individuals’ functioning, and the expenditure and workload of health services (decreasing treatment costs and wasting time).

The effectiveness of dietary treatment on psychological aspects is further confirmed by the statistically significant reduction in anxiety levels measured by SAS. A trend from moderate levels of severity to low levels at the end of TBD was observed. More importantly, a clear correlation between anxiety and bloating was found. Anxiety can likely affect the perception and symptom reporting of physiological gut-brain signals due to alterations in visceral perception [51] and colonic motility [52].

Finally, with regards to the assessment of psychophysiological activation, or the stress response, our patients showed high baseline scores well above the cut-off values. These data agree with the literature, which suggests that IBS patients experience more daily or lifelong stressful events than those with organic disorders or healthy individuals [53]. In addition, this evidence could explain why the stress levels remained above the cut-off values, even if a significant improvement in the stress condition occurred at the end of dietary treatment.

The study has some weaknesses. Firstly, the research was not based on a double-blind, controlled design. It is known that a double-blind design can be challenging to perform in dietary studies, as it cannot hide the characteristics of the diet from participants even when the researchers provide them directly. In addition, a double-blind design makes it difficult to transpose the results into real-life contexts and tailor the diet to long-term dietary conditions. It would have been worth considering a control group, especially in this type of study evaluating subjective responses. However, a pre-post design has the strength to demonstrate the beneficial effect of the intervention, even if it does not guarantee control over the variability of the disease over time. In addition, in the present study, several anthropometric and BIA parameters, beyond the symptom profile, were evaluated that are certainly not influenced by a placebo effect and are in line with each other, the symptoms, and our previous studies [13,16]. 

Secondly, in order to comprehend the complex IBS pathophysiological background, this study introduced a simplified model focused on symptoms, nutritional, and psychological changes. Consequently, the same model did not allow for verification of whether psychological factors precede abdominal bloating. Of course, many other factors could have been considered, requiring a much larger number of patients. 

## 5. Conclusions

IBS is heterogeneous and more prevalent among women than men, involving the gut-brain axis with multifaceted expressions of organic and psychological symptoms. However, the growing knowledge of its pathophysiology supports the remarkable potential of dietary therapies and alternative grains such as Tritordeum in its management. Our study is based on an experimental model that is very far from the real world and undoubtedly subject to limitations when applied to daily life. However, it is our belief that it should be pursued through further investigation so that a radical change of perspective can be undertaken regarding the therapeutic strategy of IBS towards a nutritional approach.

## Figures and Tables

**Table 1:** Irritable bowel syndrome-symptom severity scale (IBS-SSS) single items, total score, and the Bristol stool score of IBS-D patients before (pre) and after (post) 12 weeks of a Tritordeum-based diet.

Items	Pre	Post	% Reduction	*p*
Severity of abdominal pain	48.50 ± 5.30	23.78 ± 4.95	−50.97	<0.0001
Abdominal pain frequency	45.00 ± 6.58	19.00 ± 3.57	−57.78	0.0006
Severity of abdominal bloating	63.22 ± 3.97	29.44 ± 4.69	−53.43	<0.0001
Dissatisfaction with bowel habit	67.06 ± 6.17	34.72 ± 4.55	−48.22	0.0002
Interference in life in general	55.11 ± 5.83	31.11 ± 5.61	−43.54	<0.0001
Bristol Stool Score	4.9 (5.2–2.3)	4.0 (5.1–2.3)	−18.37	0.5011
Total score	278.89 ± 18.74	138.06 ± 18.07	−50.50	<0.0001

Data are expressed as means ± SEM and analyzed by the Wilcoxon matched-pairs signed rank test. All differences were considered significant at *p* < 0.05. The Bristol stool score is described as being in the median (25–75 percentiles).

**Table 2:** Anthropometric and bioelectrical impedance characteristics of IBS-D patients before (pre) and after (post) 12 weeks of a Tritordeum-based diet.

	Pre	Post	*p*
Height (m)	1.60 ± 0.01	//	//
Weight (kg)	66.09 ± 2.45	62.93 ± 2.23	0.0002
BMI (kg/m^2^)	26.04 ± 1.13	24.78 ± 1.06	0.0003
Mid-upper arm circumference (cm)	28.83 ± 0.57	27.93 ± 0.47	0.0004
Shoulder circumference (cm)	104.21 ± 1.86	101.32 ± 1.69	0.0002
Abdominal circumference (cm)	91.65 ± 2.39	88.39 ± 2.30	0.0143
Waist circumference (cm)	80.23 ± 2.77	77.31 ± 2.51	0.0014
Hip circumference (cm)	100.83 ± 2.13	97.73 ± 1.95	0.0002
PhA (degrees)	5.89 ± 0.15	6.24 ± 0.17	0.0773
BCM (kg)	23.97 ± 0.68	24.06 ± 0.56	0.3713
FM (kg)	20.56 ± 1.67	19.14 ± 1.56	0.0373
FFM (kg)	45.70 ± 1.03	43.75 ± 0.78	0.0017
TBW (L)	33.35 ± 0.79	31.79 ± 0.59	0.0027
ECW (L)	15.57 ± 0.42	14.35 ± 0.39	0.0005

BMI: Body Mass Index; PhA: Phase Angle; BCM: Body Cell Mass; FM: Fat Mass; FFM: Fat-Free Mass; TBW: Total Body Water; ECW: Extra Cellular Water. Data are expressed as means ± SEM and analyzed by the Wilcoxon matched-pairs signed rank test. All differences were considered significant at *p* < 0.05.

## Data Availability

The data that support the findings of this study are available from the corresponding author upon reasonable request.
